# Statin Therapy to Improve Outcome of COVID-19 Patients: Useful or Not Useful?

**DOI:** 10.3390/jpm12101627

**Published:** 2022-10-01

**Authors:** Bruno Mégarbane

**Affiliations:** Department of Medical and Toxicological Critical Care, Lariboisière Hospital, INSERM UMRS-1144, Paris Cité University, 75010 Paris, France; bruno.megarbane@lrb.aphp.fr; Tel.: +33-1-4995-8442

## 1. Introduction

About one year ago, the journal published a large population-based cohort study from South Korea investigating the potential benefits associated with statins at preventing severe acute respiratory syndrome coronavirus-2 (SARS-CoV-2) infection or improving the outcome of hospitalized coronavirus disease-2019 (COVID-19) patients [1]. Overall, 122,040 adults with 22,633 persons treated with statins (18.5%) and 101,697 controls (91.5%) were included. In this registry, 7780 individuals (6.4%) developed COVID-19, and 251 (3.2%) died in the hospital. Based on a propensity score matching and logistic regression analysis, statins were showed to lower the risk of SARS-CoV-2 infection (odds ratio (OR), 0.65; 95% confidence interval (95% CI), 0.60–0.71; *p* < 0.001). By contrast, statin therapy was not associated with a reduced hospital mortality among COVID-19 patients (OR, 0.74; 95% CI, 0.52–1.05; *p* = 0.09).

For one-year, numerous other clinical studies were published, and many hypotheses were suggested to explain possible interactions between statins and SARS-CoV-2 and to investigate possible clinical benefits to administer statins in COVID-19 patients. The objectives of this commentary article were to report newly published findings updating statin-attributed effects in COVID-19 patients and their underlying mechanisms.

## 2. The Relationships between Serum Lipids and SARS-CoV-2 Infection

In most studies, histories of hyperlipidemia and hypercholesterolemia were not associated with increased risk of SARS-CoV-2 infection [2,3] or with worsened outcome [4], despite contradictory findings [5]. Curiously, whether past hypertriglyceridemia might be associated with deleterious consequences on the risk or severity of SARS-CoV-2 infection remained unknown.

However, like most viral infections, marked changes in lipid metabolism and subsequently in serum lipid profile were observed in COVID-19 patients, including decreases in serum total cholesterol, HDL-cholesterol, and ApoA1 levels and elevation in serum triglycerides as compared to normal subjects [6,7]. Since lipids such as arachidonic acid and omega-3 derivatives are modulators of the inflammatory response, changes in lipid profiles result from hyperinflammatory states and are more marked in critically than mildly-to-moderately ill COVID-19 patients [8]. Of note, a decrease in HDL-cholesterol was only observed in critically ill COVID-19 patients; however, despite adjustments for age, gender, and comorbidities, conflicting relationships persisted between HDL-cholesterol levels and the risk of severe complications. In one study, mean HDL-cholesterol was reported at 1.2 mmol/L (1.0–1.5) in discharged versus 1.4 mmol/L (1.0–1.7) in hospitalized patients (*p* = 0.88) [9], but in another study, the risk of developing severe presentations was significantly associated to HDL-cholesterol (adjusted hazard ratio (HR) 2.83; 95% CI 1.19–6.71, *p* = 0.02) [10]. While total and LDL-cholesterol decreased over time and were restored on discharge, HDL-cholesterol decreased over time but was not normalized on intensive care unit (ICU) discharge [11].

## 3. The Mechanisms by Which Statins May Interact with SARS-CoV-2 Infection

Given the relationships between SARS-CoV-2 and serum lipids, the role of statins (i.e., 3-hydroxy-3-methylglutaryl-CoA (HMG-CoA) reductase inhibitors) to modulate the risk and severity of COVID-19 was questioned. Experimental studies were conducted to investigate pathways allowing statins to attenuate SARS-CoV-2 replication and the resulting cytokine storm, as well as pulmonary injuries. Multiple pathophysiological mechanisms to support such benefits were described [12].

First, direct inhibitory effects on SARS-CoV-2 cell entry and replication involving the inhibition of the viral RNA-dependent RNA polymerase and the main protease were demonstrated. Using computational blind docking analyses, statins exhibited strong although molecule-dependent affinities to critical SARS-CoV-2 structural and functional proteins, including the mutant spike proteins [13]. Stable drug–protein complexes were observed using molecular dynamics simulation, suggesting potent antiviral activities attributed to statins.

Recently, lipid rafts were identified as functional plasma membrane microdomains with tightly packed lipids enriched in sphingolipids and cholesterol and concentrating various receptors and molecules involved in pathogen recognition [14]. Lipid rafts were shown to be effective platforms for SARS-CoV-2 entry [15], due to elevated contents in signaling molecules involved in SARS-CoV-2–cell interaction such as angiotensin-converting enzyme-2 (ACE2), transmembrane serine proteases (TMPRSS), heparan sulfate proteoglycans (HSPGs), Toll-like receptors (TLRs), CD-147, and HDL-scavenger receptor B type 1 (SR-B1). Lipid rafts were shown to facilitate the attachment of the viral spike protein to the ACE-2 receptor and to allow SARS-CoV-2 to recruit key regulator proteins of the lipid metabolism [16]. Hypercholesterolemia involved in enhanced formation of lipid rafts was suggested to facilitate SARS-CoV-2’s entry into the target cells, and by contrast, drugs depleting cholesterol or altering intracellular cholesterol homeostasis such as statins were suggested to impair cell infection [15,17].

Secondly, indirect benefits of statins were attributed to their pleiotropic immunomodulatory and anti-inflammatory effects. Statins were reported to lower serum C-reactive protein (CRP) level and to moderately reduce the risk of pneumonia in healthy adults [18]. Excessive LDL-cholesterol was shown to activate the inflammasome and the release of pro-inflammatory cytokines such as interleukin (IL)-1β and IL-18 [19], explaining why dyslipidemia should be considered as a pro-inflammatory trigger. Interestingly, statins were shown to reduce serum IL-6 levels achieved by TLR4 inhibition and macrophage activity modulation. Additionally, anti-thrombosis and anti-oxidative properties consisting of the reduction in various mediators in lung tissue, such as plasminogen activator inhibitor type-1 (PAI-1), transforming growth factor-beta (TGF-β), and vascular endothelial growth factor (VEGF), were suggested to limit COVID-19-related thrombosis and resulting pulmonary fibrosis.

Using K18-hACE2-transgenic mice, statin-attributed beneficial properties were assessed in vivo [20]. Simvastatin was shown to significantly reduce SARS-CoV-2 replication, downregulate the triggered systemic inflammation, limit lung damage, and delay mortality. Simvastatin displaced ACE2 on cell membrane lipid rafts, thus affecting the course of SARS-CoV-2 infection and limiting inflammation at the infection site.

## 4. Clinical Studies Investigating the Benefits of Statins in COVID-19 Patients

Multiple clinical studies with various methodologies and flaws were conducted. Important biases were expected from simple observations since in comparison to non-statin users, statin users were supposed to be older, with more hypertension, heart failure, chronic obstructive pulmonary disease, and severe diabetes of longer duration and with more macrovascular and microvascular complications. Therefore, more accurate studies were rapidly requested.

### 4.1. Observational Studies

Pre-hospitalization statin use was shown not detrimental and not associated with an increased risk of all-cause death, ICU admission, need for invasive mechanical ventilation, and onset of myocardial infarction, transient cerebral ischemic attack, or stroke (adjusted OR, 0.82; 95% CI, 0.60–1.11; *p* = 0.82) [21]). Furthermore, accumulating retrospective findings suggested interesting benefits of pre-COVID-19 use or post-COVID-19 diagnosis administration of statins on the outcome, including large cohort studies from China [22], the US [23], the UK [24], Canada [25], and France [26], confirming the results of the South Korean registry study [1]. Reduction in the risk of death (up to 28% as evaluated in 2.9 million patients [24]) was found. Discontinuation of previous statin therapy on hospital admission once diagnosed with COVID-19 could therefore result in a worse outcome (OR, 0.65; 95% CI, 0.59–0.72; *p* < 0.001) [27]. Benefits were established in COVID-19 patients previously treated and maintained on statin therapy during hospitalization in comparison to patients in whom therapy was withdrawn on admission. Studies showed lower inflammatory markers on follow-up and reduced risk of lung injury and acute respiratory distress syndrome, reduced need for ICU admission and invasive mechanical ventilation, and reduced organ complications such as acute kidney injury, hospital-acquired pneumonia, sepsis, and in-hospital death. In most studies, benefits were determined using multivariate regression model-based and/or propensity score-matching adjusted or matched analyses (e.g., adjusted HR, 0.58; 95% CI, 0.43–0.80; *p* = 0.001 [22]; HR, 0.57; 95% CI, 0.37–0.86; *p* = 0.008 [28]; OR, 0.67; 95% IC, 0.54–0.83; *p* < 0.001 [29]; and OR, 0.52; 95% CI, 0.33–0.64, *p* < 0.001 [30]). Improved survival was also found in the critically ill COVID-19 patients with a reduction in in-hospital (HR, 0.69; 95% CI, 0.54–0.89; *p* = 0.004) and 30-day mortality (HR, 0.75; 95% CI, 0.58–0.98; *p* = 0.03) [31]. However, statins did not modify the risk of thrombosis and venous thromboembolism despite the observed benefits on survival [32]. Additionally, in some studies, benefits on the different outcomes were dissociated, such as in the nationwide Swedish cohort study including 572,695 individuals, which showed a decreased risk of death (HR, 0.86; 95% CI, 0.79–0.95; *p* < 0.001) but not of ICU admission [33]. Use of low- and moderate-intensity statins was associated with lowered risk compared with non-statin users (HR, 0.78; 95% CI, 0.71–0.86 and HR, 0.84; 95% CI, 0.80–0.89, respectively) in contrast to high-intensity statins (HR, 1.01; 95% CI, 0.86–1.18) [26]. All statin molecules reduced the risk of hospitalization, whereas pravastatin, rosuvastatin, and simvastatin, but not atorvastatin and fluvastatin, reduced the risk of death.

In less numerous studies, no benefit was found with previous statin use, including on mortality rate [34,35]. The association of statin use with lower adverse 30-day outcomes was weaker in COVID-19 patients than in non-COVID-19 patients, suggesting that statins did not exert anti-SARS-CoV-2 effects [36]. Statins did not decrease all-cause 30-day mortality by either Cox proportional hazards stratified model-based analyses (HR, 0.99; 95% CI, 0.88–1.12; *p* = 0.92) or propensity-matching analyses (HR, 0.86; 95% CI, 0.74–1.01; *p* = 0.06) [37]. Similarly, statins did not reduce the risk of ICU admission (HR, 1.02; 95% IC, 0.84–1.18; *p* = 0.71 and 0.92; 95% CI, 0.74–1.31; *p* = 0.28) nor the need for mechanical ventilation (HR, 1.06; 95% CI, 0.90–1.25; *p* = 0.47 and 1.02; 95% CI, 0.81–1.29; *p* = 0.71). It is not clear why discrepancies exist between studies and whether studies demonstrating no effects were lacking power or included patients whose vulnerabilities differed. In a Spanish cohort study, while no significant reduction in any risk was found, significant association was established between statins and reduced mortality (adjusted RR, 0.61; 95% IC, 0.43–0.86; *p* = 0.005), mechanical ventilation (adjusted RR, 0.53; 95% IC, 0.32–0.87; *p* = 0.01), and ICU transfer (adjusted RR, 0.81; 95% IC, 0.69–0.94; *p* = 0.005) in the subgroup of patients with a history of myocardial infarction or stroke [34]. In a nationwide South Korean cohort study, a protective role of previous statin use was found only in patients with past hypertension (OR, 0.40; 95% CI, 0.23–0.69; *p* = 0.009) [21]. Interestingly, in COVID-19 patients with past cardiovascular diseases, preadmission statins were associated with improved in-hospital outcome, including thrombotic complications, ICU transfer, and death, associations negated once inflammation and myocardial injury were considered [38].

A causal relationship between statin-related reduced risk of COVID-19 hospitalization and HMG-CoA reductase inhibition was suggested by Mendelian randomization studies showing an association between a higher HMG-CoA reductase expression or mediated LDL-cholesterol and a higher risk of hospitalization (OR, 1.38; 95% CI, 1.06–1.81; *p* = 0.019 and OR, 1.32; 95% CI, 1.00–1.74; *p* = 0.049, respectively) [39]. A medico-economic study established that in hospitalized COVID-19 patients, treatment with statins was both cheaper (USD 31,623 ± 20,331 versus USD 33,218 ± 25,440) and more effective (mean utility value, 1.70 ± 1.96 versus 1.71 ± 1.00) than treatment without statins [40].

Rarely, cohort studies led to deleterious results. In one study, statins increased the risk of severe COVID-19 (RR, 1.18; 95% CI, 1.11–1.27; *p* < 0.001) without altering mortality [41]. Surprisingly, the French multicenter study focusing on hospitalized diabetic COVID-19 patients (*n* = 2449 patients included and *n* = 1192 patients analyzed) showed that past statins were significantly associated with a worse outcome [42]. In unadjusted analyses, the rate of invasive mechanical ventilation and 28-day death was similar in statin versus non-statin users within both 7 (29.8% versus 27.0%; *p* = 0.1) and 28 days (36.2% versus 33.8%; *p* = 0.2) of admission. However, the mortality rate was significantly higher within both 7 (12.8% versus 9.8%; *p* = 0.02) and 28 days (23.9% versus 18.2%; *p* < 0.001). After adjustment by inverse probability of treatment weighting, statins were associated with this outcome within 7 days (OR, 1.38; 95% CI, 1.04–1.83) and death within both 7 (OR, 1.74; 95% CI, 1.13–2.65) and 28 days (OR, 1.46; 95% CI, 1.08–1.95).

### 4.2. Interventional Studies

Based on observations, pharmacological manipulation of intracellular cholesterol using statins was rapidly considered as promising to mitigate SARS-CoV-2 infection. Randomized placebo-controlled trials (RCTs) testing the benefits of statin administration in hospitalized COVID-19 patients were conducted. A first RCT conducted in mild-to-moderate COVID-19 patients showed that 40 mg atorvastatin once daily for two weeks significantly reduced serum CRP level, supplemental oxygen need, and hospitalization length [43]. By contrast, in the RESIST RCT, including mild-to-moderate COVID-19 patients, aspirin, atorvastatin, or their combination did not prevent clinical deterioration (atorvastatin: HR, 1.0; 95% CI, 0.41–2.46; *p* = 0.99 and aspirin: HR, 0.7; 95% CI, 0.27–1.81; *p* = 0.46) [44]. Another RCT conducted in ICU COVID-19 patients showed that atorvastatin did not significantly improve outcome defined as the composite of venous or arterial thrombosis, need for extracorporeal membrane oxygenation, or all-cause mortality [45]. Inconsistent with these results, an RCT showed deleterious effects of statins in hospitalized COVID-19 patients. Atorvastatin in addition to standard care delayed symptom improvement and subsequently increased hospitalization length (1.71-fold delayed remission; HR, 1.70; 95% CI, 1.22–2.38; *p* = 0.002) [46].

### 4.3. Metanalyses

At least twenty metanalyses were published, variably including only observational studies, only RCTs or all kind of studies. A metanalysis including 110,078 patients from 13 cohort studies showed that past statin therapy did not alter COVID-19-attritubted mortality risk (HR, 0.80; 95% CI, 0.50–1.28) [47] (Chow). However, post-SARS-CoV2 infection prescription of statins reduced mortality (HR, 0.53; 95% CI, 0.46–0.61), especially in patients admitted to the ICU (OR, 0.65; 95% CI, 0.26–1.64). By contrast, in a second metanalysis (*n* = 14,446 patients; 8 propensity-score matching matched studies with adjusted analysis), past statins in the non-ICU patients were associated with a reduced mortality rate (RR, 0.72; 95% IC, 0.55–0.95; *p* = 0.018 [48]. Among these in-patients receiving statins, a lower mortality was observed (RR, 0.71; 0.54–0.94; *p* = 0.030) as found in the previous metanalysis [47]. Age, gender, diabetes mellitus and past hypertension did not alter the association between previous statin use and in-hospital mortality. A third metanalysis of 35 studies confirmed that statins lowered all-cause mortality risk (HR, 0.70; 95% CI, 0.58–0.84; *n* = 21,127 patients and OR, 0.63; 95% CI, 0.51–0.79; *n* = 115,097 patients) and severe illness (OR, 0.80; 95% CI, 0.73–0.88; *n* = 10,081 patients) [49]. A fourth metanalysis including 147,824 patients clarified some issues, showing that statins did not alter mortality (unadjusted RR, 1.16; 95% CI 0.86–1.57; 19 studies) but that, when adjusting analyses, statins independently reduced the mortality rate (adjusted OR, 0.67; 95% CI, 0.52–0.86; 11 studies and adjusted HR, 0.73; 95% CI, 0.58–0.91; 10 studies) [50]. Finally, a meta-analysis limited to five RCTs (*n* = 1132 patients, 556 in the intervention group and 576 in the placebo/standard protocol group) showed no benefit of statins [51]. Length of hospital stay (mean difference, 0.25 days (−1.13 to 1.62); *p* = 0.73), ICU admission (RR, 1.72; 95% IC, 0.61–4.85; *p* = 0.31), and death (RR, 0.90; 95% IC, 0.73–1.11; *p* = 0.33) did not significantly differ.

Like isolated observational studies and RCTs, metanalyses were unable to solve discrepancies. However, decrease in all-cause in-hospital mortality in COVID-19 patients as observed in most studies supported statin use, if previously used or indicated otherwise [52].

## 5. The Issue of Statin-Attributed Adverse Effects in COVID-19 Patients

In addition to their debated effectiveness, the safety of statins might represent an issue, especially in frail and critically ill COVID-19 patients. To the best of our knowledge, no study except one [35] reported increased adverse effects such as liver cytolysis, rhabdomyolysis, and/or serum glucose dysregulation for which COVID-19 patients are considered at high-risk [31,45]. No safety issue was found in a large study in ICU COVID-19 patients [31]. However, patient monitoring should be careful. Drug–drug interactions involving cytochromes P450 3A, which metabolize statins, are highly possible [53]. In case of ritonavir-boosted nirmatrelvir prescription, substitution of simvastatin and lovastatin by pravastatin or fluvastatin and a dose reduction for atorvastatin and rosuvastatin were recommended.

## 6. Conclusions

Although this review is narrative and not systematic, our findings underline that, despite well-conducted large cohort studies, unsolved issues persist with statin prescription in the COVID-19 patient. However, considering their safety and debated benefits on outcome as suggested by robust pathophysiological evidence and multiple clinical studies, in-hospital statin administration could be considered in hospitalized COVID-19 patients and at least not stopped on admission in those previously treated, even if critically ill, given their proven benefits in cardiovascular diseases as lipid-lowering therapy.
